# Structure Based Prediction of Neoantigen Immunogenicity

**DOI:** 10.3389/fimmu.2019.02047

**Published:** 2019-08-28

**Authors:** Timothy P. Riley, Grant L. J. Keller, Angela R. Smith, Lauren M. Davancaze, Alyssa G. Arbuiso, Jason R. Devlin, Brian M. Baker

**Affiliations:** Department of Chemistry and Biochemistry and the Harper Cancer Research Institute, University of Notre Dame, Notre Dame, IN, United States

**Keywords:** structure, neoantigen, peptide, MHC, modeling, Rosetta, neural network, personalized vaccines

## Abstract

The development of immunological therapies that incorporate peptide antigens presented to T cells by MHC proteins is a long sought-after goal, particularly for cancer, where mutated neoantigens are being explored as personalized cancer vaccines. Although neoantigens can be identified through sequencing, bioinformatics and mass spectrometry, identifying those which are immunogenic and able to promote tumor rejection remains a significant challenge. Here we examined the potential of high-resolution structural modeling followed by energetic scoring of structural features for predicting neoantigen immunogenicity. After developing a strategy to rapidly and accurately model nonameric peptides bound to the common class I MHC protein HLA-A2, we trained a neural network on structural features that influence T cell receptor (TCR) and peptide binding energies. The resulting structurally-parameterized neural network outperformed methods that do not incorporate explicit structural or energetic properties in predicting CD8^+^ T cell responses of HLA-A2 presented nonameric peptides, while also providing insight into the underlying structural and biophysical mechanisms governing immunogenicity. Our proof-of-concept study demonstrates the potential for structure-based immunogenicity predictions in the development of personalized peptide-based vaccines.

## Introduction

The development of immunological therapies that incorporate peptide antigens presented to T cells by major histocompatibility complex (MHC) proteins is a long sought-after goal, particularly for cancer. Although early cancer vaccine studies relying on non-mutated shared antigens were disappointing (1), advances in sequencing and bioinformatics have led to the identification of “neoantigens” with non-synonymous mutations that differentiate tumors from healthy tissues [reviewed in (2)]. Vaccination using such neoantigens has in some cases led to promising outcomes (3, 4), and neoantigens are now also being explored as a means to improve the safety, specificity, and efficacy of other immunotherapies. A significant challenge remains, however, in identifying those mutated peptides that are immunogenic and can thus promote anti-tumor immune responses.

Following sequencing, potential neoantigens have been identified via bioinformatic approaches that predict processing and presentation by MHC proteins, and more recently, via mass spectrometry (5, 6). Mutations at anchor residues which improve the binding of a peptide to an MHC protein have been associated with immunogenicity and tumor rejection (7–9). In these cases, T cells not eliminated by negative selection may exist that efficiently recognize the neoantigen; indeed, in viruses, recent findings suggest that for peptides presented by class I MHC proteins, peptide binding affinity is the best predictor of immunogenicity (10). However, in the more common instances in which mutations occur outside of anchor residues and do not strongly impact peptide-MHC binding, T cells that efficiently recognize the wild-type peptide will have been deleted or otherwise tolerized. In these instances, an immunogenic neoantigen must possess structural and physical properties distinct enough to promote efficient recognition by T cells that ignore the wild-type peptide (i.e., the single mutation must result in a peptide that is “sufficiently different” from its wild-type counterpart to overcome self-tolerance).

However, it is becoming increasingly understood that, even after taking tolerance mechanisms into account, not all well-presented peptides are strongly immunogenic (11, 12), suggesting the existence of peptide features that influence T cell recognition independently of peptide processing and presentation. For example, recent work suggests that immunogenic peptides are enriched in hydrophobic (including aromatic) amino acids at positions often contacted by T cell receptors (TCRs) (13, 14). Efforts at incorporating features that influence T cell recognition into neoantigen prediction tools are in development (14–18), and these complement well-developed tools for predicting MHC binding (19–23). The immune epitope database (IEDB) and NetTepi servers, for example, incorporate positional enrichment of hydrophobic amino acids into class I MHC immunogenicity prediction tools (13, 18, 24). Other physicochemical features that have been considered include amino acid charge and size, wild-type and mutant sequence divergence, and sequence entropy (15, 17).

Despite these advances, the mechanisms by which physicochemical features of peptides influence TCR binding have not been widely considered. For example, enrichment in hydrophobic amino acids at potential TCR contact sites for immunogenic peptides can be interpreted in the context of protein biophysics: burial of exposed hydrophobic surface promotes protein binding through the hydrophobic effect, which is almost universally favorable, requiring only that a hydrophobic group dock into another hydrophobic environment (25–27). Burying charges, on the other hand, requires overcoming energetically expensive desolvation penalties and thus high structural precision between atoms of opposing charge (28–30). Because of this, TCRs with architectures that permit precise charge complementarity will occur less frequently than those that can accommodate a hydrophobic (or aromatic) group (31). This leads to the prediction that neoantigens whose mutations replace centrally located, charged amino acids with hydrophobic or aromatic amino acids will be immunogenic, as some studies have indeed reported (32). Likewise, introduction of charges can reduce immunogenicity, as has also been reported (33) and commonly seen in studies of T cell specificity using peptide libraries [e.g., (34)].

Yet for a peptide bound to an MHC protein, the impact of features such as exposed hydrophobic surface and charges are determined by the peptide's conformation within the binding groove, as well as the size and position of the various amino acid side chains. Thus, efforts to predict peptide immunogenicity should be strengthened by approaches that account for the structural properties of peptide/MHC complexes (8, 16, 35).

Here, we explored how considering structurally-determined physical features can improve efforts at predicting peptide immunogenicity. We developed a rapid procedure for accurately modeling large numbers of peptides bound to the common class I MHC protein HLA-A^*^0201 (HLA-A2), which was applied to a curated dataset incorporating thousands of immunogenic, non-immunogenic, and non-HLA-A2 binding peptides. We included non-HLA-A2 binding peptides as we aimed to capture both peptide binding to the MHC protein as well as TCR binding to the peptide/MHC complex, as both contribute to immunogenicity and both are governed by structural and physicochemical features. Indeed, strong TCR binding can compensate for weak peptide-MHC binding and *vice versa* (11, 12, 36); considering both elements together permits capturing the impact of both. We then trained a neural network on energetic features that are encoded not by peptide sequence, but by the modeled three-dimensional structures of the peptide/HLA-A2 complexes. The network recovered known features of immunogenic peptides such as enrichment in hydrophobicity, and, as assessed by the ability to predict CD8^+^ T cell responses, against the training data outperformed other models and prediction tools based only on sequence characteristics. Deployed against a set of HLA-A2-presented nonameric neoantigens, the network not only permitted predictions of immunogenicity, but yielded testable hypotheses about how the mutations influenced immunogenicity. From this proof-of-concept study we identify clear avenues for improvement and scale up.

## Results

### Development and Performance of a Rapid Peptide/MHC Modeling Strategy

To develop a rapid structural modeling strategy, we compiled a list of peptide/MHC structures within the Protein Data Bank (PDB). We restricted our analysis to high resolution, HLA-A2 structures presenting nonamers with good electron density. We focused on nonamers as these are the most represented in the PDB and relatively constrained in class I MHC peptide binding grooves. Additionally, nonameric, HLA-A2 data are the most represented in immunological databases. As we intended to emphasize structural differences emerging from amino acid mutations, we further narrowed our database by pairing each peptide/HLA-A2 complex with at least one other in which the peptide differed by only a single amino acid, either as a substitution or transposition. Our final database contained 53 structures presenting distinct peptide epitopes (Table S1).

**Figure 1:**
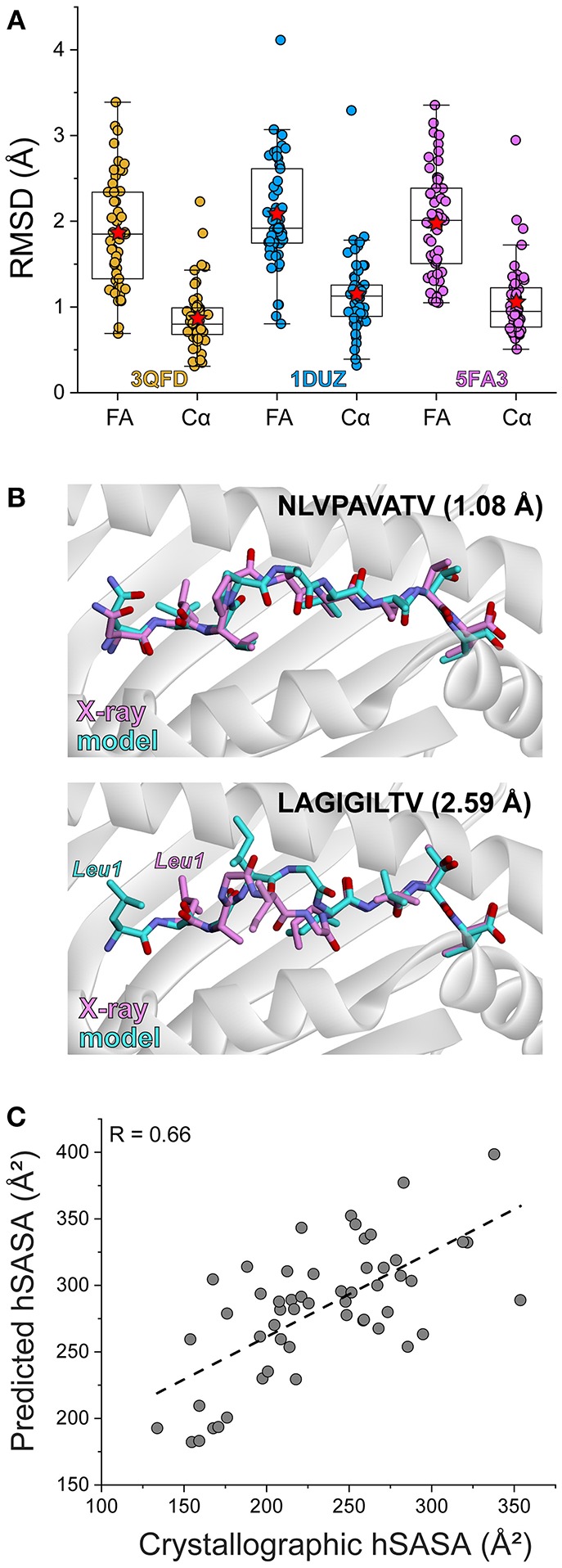
Rapid structural modeling for peptide/HLA-A2 complexes. **(A)** Modeling performance as indicated by peptide Cα and full atom (FA) RMSD values for modeled vs. crystallized peptide/HLA-A2 complexes. Boxes illustrate the 1st and 3rd quartiles, with a horizontal line at the median. Whiskers show 1.5 times the interquartile range. Red stars indicate mean values. Results for three templates (3QFD, 1DUZ, and 5FA3) are shown. The 3QFD template performed the best and was used for all further modeling efforts. **(B)** Structural images of representative models and their corresponding structures. The top shows the model of NLVPAVATV, which superimposes on the crystal structure with a full atom RMSD of 1.08 Å. The bottom shows the model of LAGIGILTV, which superimposes on the structure with a full atom RMSD of 2.59 Å. For the latter case, the leucine at position 1 forces the nonameric peptide to bind in a register-shifted decameric configuration, with the p1 leucine in the B rather than A pocket. Our modeling procedure did not permit such drastic conformational sampling. **(C)** Correlation between exposed peptide hydrophobic surface area in the models vs. the crystallographic structures. The two sets of data correlate with a R value of 0.66.

To simulate a realistic setting where many peptides need to be evaluated, we prioritized modeling speed over complexity. As has been noted previously (37), nonameric peptides bound to class I MHC proteins adopt relatively conserved backbone conformations. We therefore modeled each complex in our database by threading the desired peptide sequence into our template HLA-A2 structures, followed by Monte-Carlo-based conformational sampling and energy minimization for side chains and the peptide backbones utilizing Rosetta (38, 39). This approach required approximately 10 min per model on 2016-vintage CPU hardware. We considered three different templates to compare the effect starting coordinates had on model accuracy: HLA-A2 presenting the HTLV1 Tax_11−19_ peptide (PDF 1DUZ; peptide sequence LLFGYPVYV) (40), the MART1_27−35_ tumor antigen (PDB 3QFD; peptide sequence AAGIGILTV) (41), and a *Toxoplasma gondii* epitope (PDB 5FA3; peptide sequence GLLPELPAV) (42). These three structures were chosen based on their resolution (<1.9 Å) and variations around the nonameric backbone conformation. The modeling procedure performed similarly with all three templates, yielding full atom root mean square deviations (RMSD values) between 1.86 and 2.08 Å, and Cα RMSD values between 0.87 and 1.15 Å (Figure 1A; Table S1). Other approaches to model peptides in class I MHC binding grooves have incorporated docking, molecular dynamics simulations, protein threading, or combinations of these methods. These other methods have reported Cα or full-atom RMSD values between model and experiment within the approximate range of 1–2 Å (8, 16, 37, 43–50). Our approach thus compares favorably with or even outperforms other efforts.

Of the three templates considered, the models generated from 3QFD were the closest to the crystal structures; the average backbone RMSD of models derived from 3QFD was significantly lower (*p* = 0.0006 and 0.018 when compared to results with the 1DUZ and 5FA3 templates, respectively) (Figure 1A). Results from the 3QFD template were thus used for all further comparisons and modeling efforts.

The greatest discrepancy between modeled and actual structures was an unusual register-shifted peptide (LAGIGILTV) which, compared to the native peptide (AAGIGILTV), left the p1 (or “A”) pocket of the HLA-A2 molecule empty in the crystal structure, resulting in the nonameric peptide adopting a decameric configuration (41) (Figure 1B). Our modeling procedure was not able to sample such dramatic conformational shifts, and thus the model of this peptide resembled more traditional nonameric peptide/MHC structures.

Given recent attention on the role of exposed surface features in the immunogenicity of MHC-presented peptides, we asked how our modeling procedure recovered peptide hydrophobic solvent accessible surface area (hSASA). After comparing models and structures, the Pearson correlation coefficient between predicted and experimental hSASA was 0.66 (Figure 1C). Our rapid modeling procedure thus provides a good approximation of peptide structural properties within the binding groove of HLA-A2 and the changes that occur upon mutation.

### Experimental Test of the Peptide/MHC Modeling Strategy

**Figure 2:**
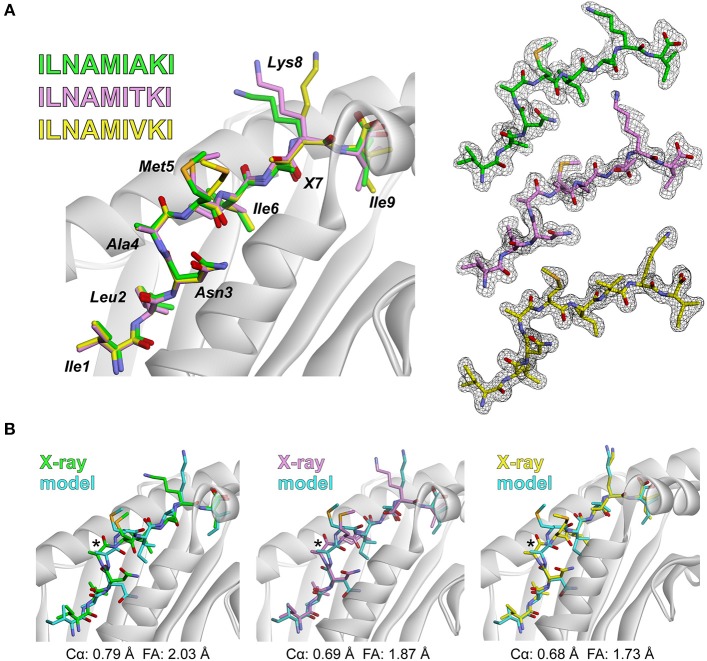
Experimental test of the peptide/MHC modeling strategy. **(A)** Structures of the ILNAMIAKI, ILNAMITKI, and ILNAMIVKI peptides in the binding groove of HLA-A2 as determined by X-ray crystallography. The peptides are colored green, pink, or yellow as indicated; this color scheme is maintained throughout the figure. The left panel shows all three structures superimposed. Peptide amino acids are indicated, with “X” used to indicate the various amino acids at position 7. The right panel shows 2F_o_-F_c_ electron density maps contoured at 1σ for peptides from each peptide/HLA-A2 structure. **(B)** Comparison of the models of each peptide/HLA-A2 complex with the crystallographic structures. For each panel, the backbone at Ala4 is highlighted with an asterisk and the Cα and full atom (FA) RMSD values for each structure/model pair are indicated.

To further test the rapid modeling procedure, we crystallized and determined the three-dimensional structures of three new peptide/HLA-A2 complexes. The peptide ILNAMIAKI is a melanoma neoantigen identified in a recent study (51). We determined the structure of ILNAMIAKI bound to HLA-A2, as well as those of the corresponding wild-type peptide ILNAMITKI and another single amino acid variant, ILNAMIVKI (Table 1). We also subjected the three complexes to the modeling procedure described above. In the structures, the peptides all adopt the typical nonameric conformation, with a bulge initiating at Asn3 and continuing through Ile6. There are no systematic changes in response to the differences at position 7. The sidechains of Met5 and Lys8 extend away from the peptide backbone, with some variations in torsion angles across the three structures (seen as well in the multiple copies in the asymmetric units for the structures with ILNAMITKI and ILNAMIAKI) (Figure 2A). The models compared well with the crystallographic structures. One discrepancy was found at the backbone of Ala4, which impacted the geometry of the subsequent Met5 side chain. Nonetheless, the position and extension of the Met5 side chain were well captured, as was the similarly extended Lys8 side chain (Figure 2B). The peptide Cα RMSD values between structures and models were between 0.7 and 0.8 Å, and the full atom RMSD values were between 1.7 and 2.0 Å. These values are consistent with the results found when comparing modeled to previously determined experimental structures (Figure 1A) and confirm that our modeling scheme can reproduce major structural features of peptide/HLA-A2 complexes.

**Table 1:** X-ray data collection and refinement statistics.

	**ILNAMIAKI/HLA-A2**	**ILNAMITKI/HLA-A2**	**ILNAMIVKI/HLA-A2**
Data collection			
Resolution (Å)^*^	30.89–2.15 (2.23–2.15)	41.82–1.90 (1.97–1.90)	31.57–1.79 (1.84–1.79)
Space group	P1	P1	P 21 21 21
Unit cell dimensions (Å)	50.68, 63.67, 75.18	58.43, 84.11, 85.36	49.56, 74.64, 122.85
Unit cell angles (°)	81.47, 75.85, 77.23	90.01, 90.06, 90.02	90, 90, 90
Unique reflections^*^	47,203 (4,655)	123,624 (11,774)	42,624 (2,553)
R-merge	0.205 (0.684)	0.096 (0.290)	0.167 (0.203)
I/σ^*^	5.7 (1.3)	10.8 (4.3)	22.2 (2.6)
Data completeness^*^	97.9% (96.3%)	96.7% (92.6%)	97.6% (87%)
Refinement			
R-work, R-free	0.19, 0.23	0.16, 0.19	0.17, 0.20
R-free test set	4711 (10.00%)	12476 (10.1%)	1955 (4.59%)
Wilson B-factor (Å^2^)	24.0	15.1	22.6
Total number of atoms	6,853	14,303	3,626
Bond lengths RMSD (Å)	0.005	0.003	0.008
Bond angles RMSD (°)	1.02	0.661	0.927
Ramachandran (favored, allowed, outlier)	98%, 2%, 0%	98%, 2%, 0%	98%, 2%, 0%
PDB ID Code	6PTB	6PTE	6OPD

* *Values in parentheses are statistics for the highest resolution shells*.

### Collecting a Peptide Dataset to Relate Peptide Structural Features to CD8^+^ T Cell Responses

To test whether consideration of structural features could lead to improved immunogenicity predictions, we developed a peptide database that contains immunogenic and non-immunogenic peptides. We again emphasized nonameric, HLA-A2 restricted peptides for consistency with our modeling strategy and as data for HLA-A2-presented nonamers is most represented in various immunological databases.

While the IEDB has records for immunogenic peptides, it contains limited data on peptides that are poorly immunogenic yet still well-presented by MHC proteins. To account for such peptides, we relied on lists of peptides identified via proteomic analyses of human HeLa cells (52, 53), yielding a dataset of 2756 nonameric, HLA-A2-presented self-peptides. While this dataset will necessarily include peptides that would be efficiently recognized by TCRs, we rationalized it would be dominated by peptides that bind well to the MHC protein but are not well-recognized (i.e., in a host, peptides that might pass positive selection but not would not drive negative selection). To this set of self-peptides, we added 155 well-characterized immunogenic peptides listed in the IEDB, selected by filtering for HLA-A2-presented human nonamers with IFN-γ ELISPOT response frequencies of 50 or higher in order to minimize false positives. The immunogenic peptide dataset primarily included epitopes from viral sources, although humans and other organisms were also represented (Table S2). The dataset included multiple amino acid variants of various peptides, which we rationalized would be important when aiming to predict the immunogenicity of mutant peptides and their wild-type counterparts.

We completed our dataset by adding 1044 HLA-A2-incompatible peptides selected from IEDB training sets. Incorporating non-HLA-A2 binding peptides ensured that our efforts addressed both TCR and MHC binding, as both directly contribute to immunogenicity and are dependent upon structure-determined energetic features. Accounting for both TCR and MHC binding together is necessary for predicting immunogenicity, as a peptide that binds weakly to an MHC protein could still prove immunogenic by possessing optimal features for TCR binding and *vice versa* (11, 12, 36). Moreover, peptide mutations can influence both TCR and MHC binding simultaneously, as seen with differential T cell recognition of some “anchor fixed” shared tumor antigens (41, 54).

**Figure 3:**
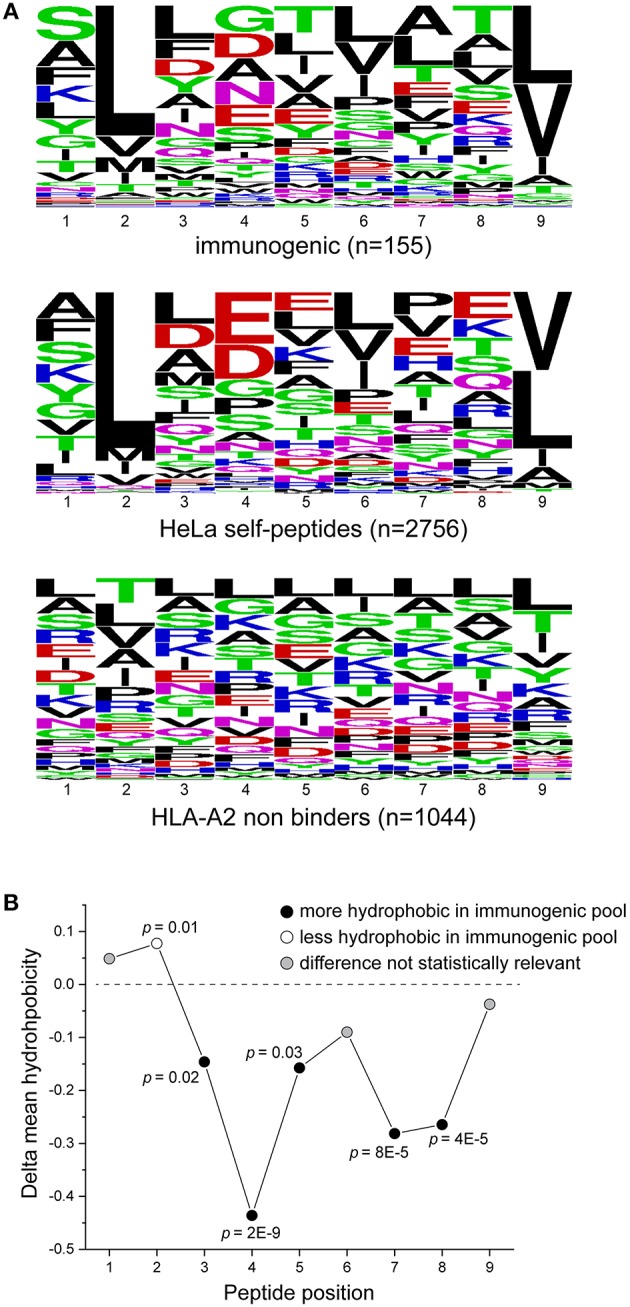
Characteristics of peptides in the training set. **(A)** Sequence logos of immunogenic peptides (top), HeLa self-peptides (middle), and HLA-A2 non-binding peptides (bottom). **(B)** Comparison of the hydrophobicity of each peptide position in the immunogenic and self-peptide datasets (shown as the difference between the immunogenic and self-peptide datasets) as determined using the Wimley-White hydropathy index. Values less than zero (below dashed line) indicate greater hydrophobicity in the immunogenic dataset. *P*-values are indicated where the differences are statistically significant.

Amino acid distributions for the immunogenic, HeLa, and HLA-A2-incompatible peptides are shown in Figure 3A. To further ask if our dataset reflected previously noted distinctions between immunogenic and non-immunogenic peptides, we evaluated the hydrophobicity of the peptides in the immunogenic and HeLa self-peptide pools. Using the Wimley-White interface hydropathy index (55), we determined the mean hydrophobicity for each peptide position in the two pools. Comparing the results for the two showed that certain positions across the peptides were statistically more likely to be more hydrophobic in the immunogenic than the HeLa self-peptide pool, with the most pronounced differences at positions 4, 7, and 8 (Figure 3B). These results, including the distinctiveness of positions 4, 7, and 8, are consistent with previous observations (13, 14) and support the conclusion that our peptide pools appropriately encompass both immunogenic and non-immunogenic peptides.

### A Neural Network to Predict Immunogenicity From Structure-Derived Parameters Outperforms Other Approaches

Using our structural modeling procedure and the database of peptides, we next constructed an artificial neural network to predict the immunogenicity of nonameric peptides bound to HLA-A2, relying on structural and energetic features determined from three-dimensional models as the network inputs. Using our rapid modeling scheme, we first generated structural models of all 3,955 peptide/HLA-A2 complexes. To describe the conformation-dependent physical properties of the peptides in the binding groove, we used the 18 terms in the Talaris2014 energy function to evaluate the energy of each modeled peptide/HLA-A2 complex (39, 56). The terms, listed in Table S3 and described in Alford et al. (56), account for features such as energies of attraction, repulsion, and solvation; energies of side chain and backbone hydrogen bonds; and energies and probabilities of side chain and backbone conformations. We also selected nine terms from the same energy function for all nine positions in the peptide, choosing terms that emphasized atomic-level features and avoiding those descriptive of particular amino acids (e.g., tyrosine planarity). To the nine amino-acid level terms, we also added total and hydrophobic solvent accessible surface areas. Overall, 117 terms that describe each modeled peptide/HLA-A2 complex were used as network inputs.

As with previous efforts in predicting immunogenicity, we used a binary classification system for each peptide in our dataset, classifying peptides identified from the IEDB as immunogenic (score of 1) and the HeLa and non-HLA-A2 binding peptides as non-immunogenic (score of 0). The network output is thus a score, from zero to one, indicating the degree of confidence in immunogenicity.

**Figure 4:**
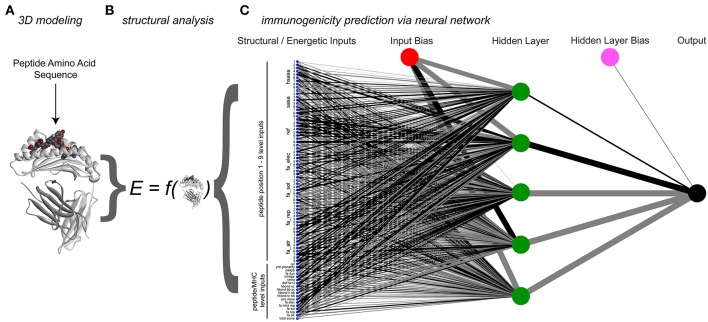
Process and architecture of the structure-based immunogenicity neural network. **(A)** The process begins with a peptide sequence, which is used to generate a model of the peptide/HLA-A2 three-dimensional structure. **(B)** Analysis of the modeled structure yields energetic and topographical information, which are used as inputs for the structure-based immunogenicity neural network. **(C)** Trained neural network architecture, with 81 structure-derived inputs shown on the left (seven for each peptide position, 18 for the overall complex). A single hidden layer is present with five hidden neurons, along with two constant bias nodes. Black lines give positive weights, gray lines negative weights, with line width indicating weight magnitude.

**Figure 5:**
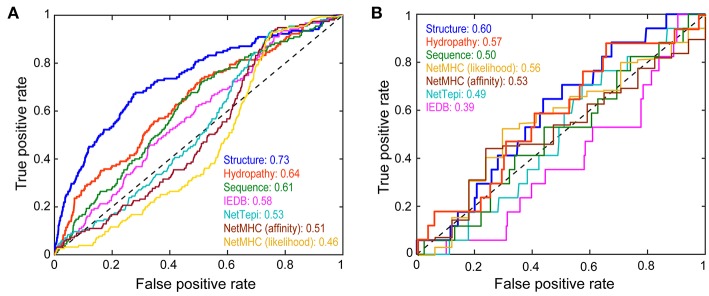
Performance of the structure-based neural network in categorizing peptide immunogenicity. **(A)** Performance of the structure-based neural network compared to control sequence-only and hydropathy-only neural networks, the IEDB and NetTepi immunogenicity prediction servers, and NetMHCpan 4.0 in evaluating the training data as demonstrated by a receiver operating characteristic curve. The area under the curve (AUC) for each approach gives the probability that the approach will more favorably score an immunogenic peptide than a non-immunogenic peptide. The structure-based neural network performed the best. **(B)** Against a neoantigen dataset of 291 nonameric peptides not used for training the structure-based network performed less favorably but still outperformed the other approaches.

In developing the neural network, we used a nested 5-fold cross-validation procedure that eliminated redundant terms. The final model consisted of the 18 terms for the entire peptide/MHC complex and seven for each amino acid in the peptide, yielding 81 terms for network inputs, with five hidden neurons and two constant bias nodes (Figure 4; Table S3). The average cross-validated area under the curve (AUC) in a receiver operating characteristic (ROC) plot was 0.69 (the AUC values in the ROC plots predict the probability that the neural network will more favorably score an immunogenic peptide compared to a non-immunogenic peptide). After training with the entire dataset, the final neural network classified all peptides used with a total AUC of 0.73 (Figure 5A).

To assess the added value of structural and energetic information, we developed a control neural network trained on the same 3,955 peptides but encoded by a sparse matrix that considered only peptide sequence. In this head-to-head comparison, the structurally-parameterized network outperformed the sequence-only network (AUC of 0.73 vs. 0.61), demonstrating conclusively that incorporating structural and energetic features improves predictions compared to considering sequence alone. As a further control, we developed another sequence-based network that considered only amino acid hydropathy values. The hydropathy network outperformed the sequence-only network (AUC of 0.64 vs. 0.61), but still did not match the performance of the structurally-parameterized network.

For comparison to other tools, we evaluated the same 3,955 peptides with the IEDB immunogenicity and the NetTepi immunogenicity prediction tools (13, 18, 24). IEDB classified the peptides with an AUC of 0.58, whereas NetTepi yielded a value of 0.54. Although not designed to predict immunogenicity, peptide-MHC binding predictions are often used in this fashion when assessing putative neoantigens, largely due to experience from viral antigens (10). Consistent with earlier findings (7), predictions using NetMHCpan 4.0 (19) did not perform well, yielding AUC values of 0.51 in affinity mode and 0.46 in ligand-likelihood mode. Overall then, whether compared with a simpler sequence-based neural network, a network capturing solely amino acid hydrophobicity, existing sequence-based immunogenicity tools, or predictions of peptide-HLA-A2 binding affinity, the structure-based network performed the best in predicting immunogenicity.

### Significance of Structure-Derived Energetic Network Inputs for Classifying Immunogenicity

Although interpreting the weights of inputs used within a neural network is difficult due to the complexity and non-linear nature of the models, the weights of structural features used within the model can provide clues to their contributions in the evaluation of immunogenicity. For MHC binding, the structure-based network considered the impact of anchor residues 2 and 9 by assessing terms such as favorable van der Waals interactions at these positions in order to quantify how compatible an epitope was with HLA-A2. The network also focused on the interactions surrounding peptide position 3, likely considering peptide-MHC interactions in this tightly packed region of the HLA-A2 binding groove.

Consistent with the hypothesis that solvent exposed residues provide information regarding peptide immunogenicity by promoting TCR binding, the network emphasized hydrophobic SASA. Notably, the weights for hydrophobic SASA and hydrophobic solvation energy values at positions 5, 7, and 8 were in the top 10% of all weights in the neural network. These positions are typically “TCR facing” in HLA-A2-presented nonameric peptides. Indeed, in the structural models used for evaluating the modeling, positions 5, 7, and 8 had high degrees of solvent exposure, and crystallographic structures of TCRs bound to nonameric peptide/HLA-A2 complexes show that these positions on average bury more than 80% of their exposed surface upon receptor binding (Figure S1).

One notable result from our analysis was that, excluding the non-HLA-A2 binding peptides, the average computed energies of the immunogenic complexes (as determined by the Talaris2014 total energy score used in the structural modeling) was higher than the non-immunogenic complexes. Although the difference was small (average of −560 Rosetta energy units for immunogenic complexes vs. −562 for non-immunogenic complexes), the energy reflects the entire peptide/MHC complex, of which the peptide is only approximately 2% by mass. Scoring only the peptides (in the context of the binding groove) recapitulated this trend (average of 11 Rosetta energy units for immunogenic peptides vs. 9.6 for non-immunogenic), and the difference was statistically significant (*p* = 0.0017). We believe this to be an indicator of how structure and energy can influence the immunogenicity of neoantigens: amino acid substitutions that impart a higher energy onto a peptide/MHC (for example, by removing exposed charges and/or increasing exposed hydrophobic surface area) yield ligands that have more energy to release upon TCR binding, translating into stronger binding affinities.

As a separate test of this hypothesis, we computed the total and hydrophobic SASA for the models of the immunogenic and non-immunogenic peptides (again excluding the non-HLA-A2 binding peptides). Although the difference in total SASA was insignificant, the exposed hydrophobic solvent accessible surface area of the immunogenic peptides was higher than the non-immunogenic peptides (244 Å^2^ vs. 224 Å^2^; *p* = 9 × 10^−7^). Exposing hydrophobic surface to water raises free energy via the hydrophobic effect, with widely used estimates relating 1 Å^2^ of exposed hydrophobic surface to 25–50 cal/mol in free energy (57–59). The surface area analysis is consistent with the results from the Rosetta scoring and supports our interpretation that immunogenicity can arise from peptide substitutions that yield higher energies and subsequently stronger TCR binding affinities.

### Testing Performance on Data Not Used in Training

Our structure-based neural network outperformed sequence-based tools when classifying the training data. Ideally, large test sets of neoantigens would be available for further evaluation. Unfortunately, the number of well-categorized neoantigens is still small, and further reduced by our restriction on nonamers presented by HLA-A2. A recent survey identified ~1,400 potential neoantigens (60), 291 of which were nonameric peptides presented by HLA-A2 (Table S4). Of these, 17 were reported as immunogenic. Although the numbers are small, in evaluating these peptides, the structure-based neural network outperformed sequence-based approaches when considering the impact of a mutation on immunogenicity (Figure 5B). Performance was only marginally favorable (AUC of 0.60), but again the dataset is small, and these epitopes are not curated or vetted to the same extent as those recorded in the IEDB.

### Evaluation of Select Neoantigens and Their Wild-Type Counterparts

To illustrate how structural information can help inform the determination of immunogenicity and provide hypotheses for testing and improving our approach, we examined structural models of mutant peptides and their wild-type counterparts, choosing select epitopes that could demonstrate the principles encoded by our structure-based assessments as well as highlight areas for improvement.

**Figure 6:**
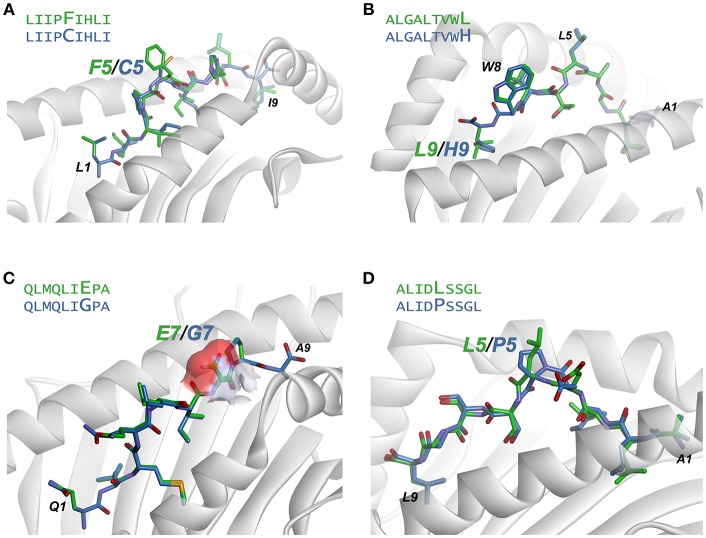
Examination of modeled structures of select neoantigens and their wild-type counterparts. **(A)** The neoantigen LIIPFIHLI substitutes a phenylalanine for a cysteine at position 5. The position 5 side chain is predicted to extend from the top of a bulge in the peptide, and the mutation results in an increase in exposed hydrophobic surface of 90 Å^2^. **(B)** The neoantigen ALGALTVWL substitutes a leucine for a histidine at position 9, “fixing” the second primary anchor residue and improving peptide binding to HLA-A2. The leucine at position 5 and tryptophan at position 8 are predicted to extend up from the peptide backbone to form interactions with T cell receptors. **(C)** The neoantigen QLMQLIEPA substitutes a glutamate for a glycine at position 7. The glutamate 7 side chain in the mutant peptide is predicted to be fully exposed, increasing exposed charged surface area as indicated by the surface area representation for the position 7 side chain. **(D)** The neoantigen ALIDLSSGL replaces a proline with a leucine at position 5 of the peptide. No conformational consequences are predicted for the mutation.

The LIIPFIHLI epitope was identified in a study of heterologous T cell recognition of melanoma neoantigens, and incorporates a cysteine to phenylalanine substitution at position 5 (32). The structural models show the position 5 side chain to be almost fully extended, with the phenylalanine mutant resulting in the exposure of an additional 90 Å^2^ of hydrophobic surface area, which could promote stronger TCR binding due to the hydrophobic effect (Figure 6A). The structure-based neural network indeed predicted the mutation would improve immunogenicity, with an increase in score of 0.14.

ALGALTVWL was identified in a study of neoantigens in breast cancer (61). This epitope replaces an unfavorable histidine in the primary HLA-A2 anchor residue at position 9 with a preferred leucine, and thus improves peptide binding to the MHC protein. Demonstrating how our approach captures not only TCR binding to the peptide/MHC complex but also peptide binding to MHC, the neural network correctly predicted the leucine variant to be more immunogenic than the wild-type peptide with the position 9 histidine, with an increase in the immunogenicity score of 0.19. Structurally, the model predicts the leucine at position 5 and tryptophan at position 8 to be solvent exposed, likely responsible for forming interactions with neoantigen-specific TCRs (Figure 6B).

QLMQLIEPA was identified in a large-scale study of neoantigens in melanoma (62). The peptide substitutes a glutamate for a glycine at position seven of the peptide. The neural network predicted the mutated peptide would be less immunogenic than the wild-type peptide, with a change in score of −0.16. Indeed, no T cell responses were identified with QLMQLIEPA. The modeling indicates the new glutamate would be fully exposed (Figure 6C), which as discussed above would likely require close charge complementarity by an incoming TCR, consistent with the reduced likelihood of immunogenicity.

Lastly, ALIDLSSGL was identified in the same study as QLMQLIEPA (62). The peptide incorporates a leucine to a proline substitution at position 5 of the peptide. The neural network predicted the neoantigen to be more immunogenic than the wild-type peptide; however, no T cell responses were identified with the peptide. This false positive may be due to a conformational impact of the proline mutation: the structural modeling predicts that both mutant and wild-type peptides adopt very similar pathways through the HLA-A2 binding groove, with a minor impact on the position of Asp4 (Figure 6D). However, a proline substitution could impact the peptide backbone in a fashion not captured by our modeling scheme, possibly indicating a need for more exhaustive conformational sampling in the structural modeling as discussed below.

## Discussion

Identifying immunogenic peptides in cellular immunity remains a challenge, particularly in the development of personalized “neoantigen” vaccines based on individual tumor genomes. Here, we tested the hypothesis that immunogenicity predictions for peptides presented by class I MHC proteins could be improved by considering features encoded by the structure of the peptide/MHC complex, rather than by features of the peptide amino acid sequence alone. Our hypothesis was predicated on the notion that immunogenicity is influenced by both peptide binding to the MHC protein as well as TCR binding to the peptide/MHC complex. Prediction methods for the former are well-developed, whereas prediction methods for the latter are in their infancy. Some peptide features that possibly promote TCR binding have been identified (13–17), but we suggest these and other important features are best interpreted, and ultimately predicted, by examining structures and their physicochemical properties and energies. Identifying these features and the magnitudes required are particularly important for tumor neoantigens, as the bar for establishing “difference from self” for neoantigens is higher than for antigens from viruses or other pathogens due to the various tolerance mechanisms that limit self-reactivity.

To explore our hypothesis, we developed a rapid and accurate procedure for modeling the structures of nonamers bound to the class I MHC protein HLA-A2. Our procedure performed well-compared to previously published methods and was suitably rapid for use with large peptide databases. Following this, we assembled a database of immunogenic and non-immunogenic peptides, including in the latter HLA-A2-incompatible peptides predicted to be very weak binders. We used the structural modeling procedure to model the nearly 4,000 peptide/HLA-A2 complexes in this database. We then trained an artificial neural network for predicting immunogenicity that relied on energetic features determined by the structures. As potential terms to be incorporated, we included features such as van der Waals interactions, hydrophobic solvation, Coulombic potentials, hydrogen bond energies, side chain rotamer energies, as well as solvent accessible surface areas. We designed our approach to capture both peptide binding to the MHC protein as well as TCR binding to the peptide/MHC, as both are determined by structural and physicochemical features, and in determining immunogenicity, strong TCR binding can compensate for weak peptide-MHC binding and *vice versa* (11, 12, 36).

Our structural approach outperformed other prediction tools, including comparisons with sequence-based neural networks trained on the same peptide datasets. Our model also outperformed publicly available immunogenicity prediction tools, as well as predictions based on peptide-MHC binding affinity. Beyond immunogenicity prediction, an important added benefit of our approach is the availability of structural models to aid in interpreting results. The utility of these models is found when comparing results for mutant peptides and their wild-type counterparts, as the models can indicate the types of structural alterations that impact TCR recognition of a mutated vs. wild-type peptide/MHC complex. In cases in which the mutation improves immunogenicity by enhancing peptide binding to the MHC protein, the models still provide indications about which peptide features may be important for TCR recognition.

A key observation that emerged from our analysis is that, excluding non-HLA-A2 binders, immunogenic peptides possessed higher total energies than non-immunogenic complexes. We believe this result is an important indicator of how structure and energy can influence immunogenicity beyond simply enhancing peptide affinity for the MHC protein: amino acid substitutions that impart a higher energy onto a peptide/MHC complex yield ligands that have more energy to release upon TCR binding, thus translating into stronger TCR binding affinities. Higher energy would, for example, be imparted by removing exposed charges or increasing exposed hydrophobic surface area. Given a compatible TCR, this high energy would be released upon binding, contributing to a favorable TCR-peptide/MHC binding free energy, i.e., better TCR binding affinity. From this interpretation, neoantigens have a greater likelihood of being immunogenic not simply when they are chemically or structurally “different” from a corresponding wild-type peptide, but different in ways that promote strong TCR binding. In addition to helping explain immunogenicity, this interpretation connects immunogenicity to well-understood physical attributes of biomolecular recognition, and is consistent with studies on the composition and structural properties of protein-protein interfaces and how they differ from other protein surfaces, as well as the properties of “hot spots” in protein-protein interfaces (63, 64).

Although we consider our results promising, improvements are necessary before a structural modeling/energetic scoring methodology can be widely deployed. Our rapid modeling procedure, while matching or even exceeding the performance of previous approaches, did not capture all the observed structural changes that occur in response to peptide modification. We expect that incorporating more exhaustive conformational sampling will yield superior models. Although this will increase computational time, this impact will be offset by ongoing improvements in computing hardware and sampling methods. Growth in the number of crystallographic structures of peptides bound to class I MHC proteins, particularly structures of closely related peptide pairs, will help benchmark the accuracy of structural modeling. Our study was limited to nonamers and HLA-A2, primarily because of the large amount of structural and immunological data available for nonamer/HLA-A2 complexes. In the absence of more structural data, extension to peptides of other lengths and other HLA haplotypes will call for even more sophisticated modeling.

Another area for methodological improvements is in the energy functions and other terms used to evaluate peptide/MHC models. We relied upon an energy function and set of terms frequently used in the analysis and design of protein structures. As with modeling procedures, more complex means to assess protein structures and energies are available, and these undergo regular refinements. Incorporation of additional or more sophisticated energetic terms (e.g., electrostatic surface potentials, more accurate approaches to computing solvation energies, and consideration of changes in peptide flexibility that occur upon mutation) could thus also be explored.

Attention should also be focused toward generating datasets that can be used to train models aiming to predict peptide immunogenicity (65). Epitopes with verified, strong immune responses can be found in the IEDB as we relied upon here, and efforts such as the Cancer Antigenic Peptide Database aim to tabulate immunogenic neoantigens (66). However, experimentally validated immunogenic neoantigens remain rare, and some efforts aimed at building CTL epitope databases prioritize peptide processing and MHC presentation over T cell recognition (67). Additionally, prediction tools also require knowledge of non-immunogenic epitopes, particularly those that bind well to MHC proteins yet do not favor TCR binding. We relied on a list of self-peptides which we hypothesized would be dominated by such epitopes, but to some extent would also include ones that are well-recognized by TCRs. This would include not only self-peptides that would drive negative selection, but as our peptide list was derived from immortalized HeLa cells (52, 53), it also likely includes various HPV epitopes and associated neoantigens. The fact that we recovered previously identified positional differences in hydrophobicity between immunogenic and non-immunogenic peptides suggests that the influence of such peptides in our self-dataset is small. However, a better accounting of peptides (ideally derived from healthy tissues) which bind well to class I MHC proteins yet do not promote strong immunogenicity when tested across multiple T cell populations is needed.

Lastly, our prediction efforts were centered on the ability to elicit strong CD8^+^ T cell responses. Eliciting CD8^+^ T cell responses remains a goal of peptide-based vaccine efforts for both pathogens and cancer (68), and CD8^+^ T cell activity is associated with antitumor immunity (69). However, additional factors will influence successful peptide-based vaccines. A deeper understanding of the strengths and types of responses associated with successful antitumor immunity will further allow prediction efforts to improve.

In conclusion, we have explored the potential for large-scale structure-based modeling and energetic scoring for predicting peptide immunogenicity, with an emphasis on cancer neoantigens. Our approach outperformed other approaches and although it is a proof-of-concept, the avenues for improvement are clear and actionable. The structural modeling allows for insights into immunogenicity lacking from other approaches. Furthermore, because it is fully atomistic, the approach can grow to incorporate complexities not addressable via sequence considerations alone, such as those arising from peptides incorporating post translational modifications or non-standard amino acids.

## Materials and Methods

### Structural Modeling of HLA-A2 Presented Peptides

Structural modeling of peptide/HLA-A2 complexes was performed with PyRosetta using the Talaris2014 energy function (38, 39). The desired peptide sequence was computationally introduced into HLA-A2, using PDB IDs 1DUZ, 3QFD, and 5FA3 as templates (40–42). This was followed by 50 Monte Carlo-based simulated annealing sidechain and peptide backbone minimization steps using the LoopMover_Refine_CCD protocol, generating 10 independent decoys per peptide from each starting template. The large number of resulting packing operations introduced some minor variability when scoring the models. Therefore, the unweighted score terms for the three lowest scoring trajectories were averaged and used for neural network inputs. Solvent accessible surface area calculations were performed with PyRosetta and Discovery Studio using a 1.4 Å radius probe. The modeling procedure is available as Supplementary File 1.

### Dataset Collection

Our structural database for evaluating modeling strategies consisted of high resolution (<3.0 Å) nonameric peptide/HLA-A2 structures within the PDB. Structures in this dataset were selected for strong electron density as determined by visual inspection using Coot for calculating 2F_o_-F_c_ density maps (70). Our final database contained 53 structures presenting different peptide epitopes.

The neural network training set contained 3,955 nonameric peptides collected from published sources. For self-peptides categorized as non-immunogenic we used lists of peptides identified via mass spectrometry analysis of human HeLa cells transfected with soluble HLA-A2 (52, 53). HLA-A2 incompatible nonamers were obtained from IEDB training sets (24). Immunogenic peptides were selected from IEDB to ensure quality of data and minimize false positives by selecting only HLA-A2-restricted nonamers with a positive IFNγ ELISpot with a response frequency starting at 50. The test dataset was derived from a recent review of neoantigens (60), again selecting only nonamers presented by HLA-A2 for evaluation, resulting in a dataset consisting of 291 candidate neoantigens.

## Artificial neural network training

Two-layer feed-forward networks were trained with the scaled conjugate gradient back-propagation trainscg tool in MATLAB 2017b. Training and evaluation of neural network architectures was performed using a nested 5-fold cross-validation procedure (23). The peptides in the training dataset were randomly split into five sets of training, validation, and test data. The splitting was performed such that all sets had approximately the same distribution of non-binding, self, and immunogenic peptides. With the binary classification of immunogenic or non-immunogenic (with non-immunogenic incorporating self and non-binding peptides), the training data were used to perform feed-forward and back propagation. The validation set defined the stopping criteria for the network training, and the test set evaluated performance via AUC. Sets were rotated to ensure each was used in training, validation, and testing. To maintain an equal distribution of classifiers and eliminate bias for non-immunogenic peptides, immunogenic peptides in the training sets, but not testing or validation sets, were randomly oversampled.

The structure-based neural network architecture used was a conventional feed-forward network with an input layer containing 80–117 neurons, one hidden layer with 1–10 neurons, and a single neuron output layer. The neurons in the input layer describe structural and structure-derived energetic features of the nine amino acids in the peptide sequence, with each amino acid represented by up to 11 neurons. The remaining 18 neurons describe global structural and structure-derived energetic features of the entire peptide/HLA-A2 complex. The structural and energetic features were those that comprise the Talaris2014 energy function (39) or derived from the structure as listed in Table S3 [described in (56)]. For each of the five training and test sets, a series of network trainings were performed each with a different number of hidden neurons (2, 3, 4, 6, 8, and 10) and a different number of input neurons. Finally, a single network with the highest test performance was selected.

For control networks that considered peptide sequence or amino acid hydropathy values, we encoded peptide sequences in 20 × 9 sparse matrices encoding peptide sequence or 1 × 9 matrices containing Wimley-White hydropathy values (55) corresponding to the amino acid at each position. These matrices were used to train networks of the same architecture (except they relied on 180 or 9 input nodes) subject to the same cross validation procedure.

### Protein Crystallization and Structure Determination

Purified complexes of ILNAMIVKI, ILNAMIAKI, and ILNAMITKI with HLA-A2 were generated by refolding recombinant heavy chain and β_2_-microglobulin from bacterially-produced inclusion bodies according to standard procedures (71), followed by purification using anion exchange and size-exclusion chromatography. Peptides were synthesized commercially by AAPTEC at >90% purity. Crystals of the ILNAMIVKI complex were grown by hanging-drop vapor diffusion at 23°C in 20% PEG 8000, 100 mM MES pH 6.5, 200 mM magnesium acetate. Crystals of the ILNAMITKI complex were grown at 23°C in 20% PEG 3350, 100 mM HEPES pH 7.5. Crystals of the ILNAMIAKI complex were grown at 4°C in 15% PEG 3350, 100 mM MES pH 6.5. Crystals were harvested and cryoprotected in ~15% glycerol and ~85% mother liquor and then immediately frozen in liquid nitrogen.

Data for the ILNAMIVKI complex were collected at the 22ID beamline at the Advanced Photon Source at Argonne National Laboratories. Data for ILNAMITKI and ILNAMIAKI complexes were collected at the 23ID-D beamline at the Advanced Photon Source. For the ILNAMIVKI and ILNAMITKI structures, data integration and scaling were performed using the HKL2000 suite. Integration and scaling of the ILNAMIAKI data were performed with DIALS (72). The structures were solved by molecular replacement using Phaser in PHENIX (73), with PDB 3PWL used as a search model for ILNAMIVKI and ILNAMIAKI (74), while 1TVH was used as a search model for ILNAMITKI (75). Peptides were deleted from search models prior to molecular replacement. Multiple steps of restrained refinement were performed using PHENIX Refine (76). Evaluation of models and fitting to maps were performed using Coot (70). MolProbity was used to evaluate structures during and after refinement (77).

## Data Availability

The experimental structural data generated for this study can be found in the Protein Data Bank under accession numbers 6OPD, 6PTB, and 6PTE.

## Author Contributions

TR developed the modeling, scoring, and prediction approaches. GK analyzed data and performed control analyses. JD analyzed data and compared to published results. GK, AS, LD, and AA performed X-ray crystallography. BB oversaw and directed the research. The manuscript was written and edited by all authors.

### Conflict of Interest Statement

The authors declare that the research was conducted in the absence of any commercial or financial relationships that could be construed as a potential conflict of interest.
