# Supplementary material for: Structure Based Prediction of Neoantigen Immunogenicity
Source: Front Immunol. 2019 Aug 28;10:2047. doi: 10.3389/fimmu.2019.02047 (PMC6724579; doi:10.3389/fimmu.2019.02047)
Supplement: Supplementary file 1 [file Data_Sheet_1.PDF]

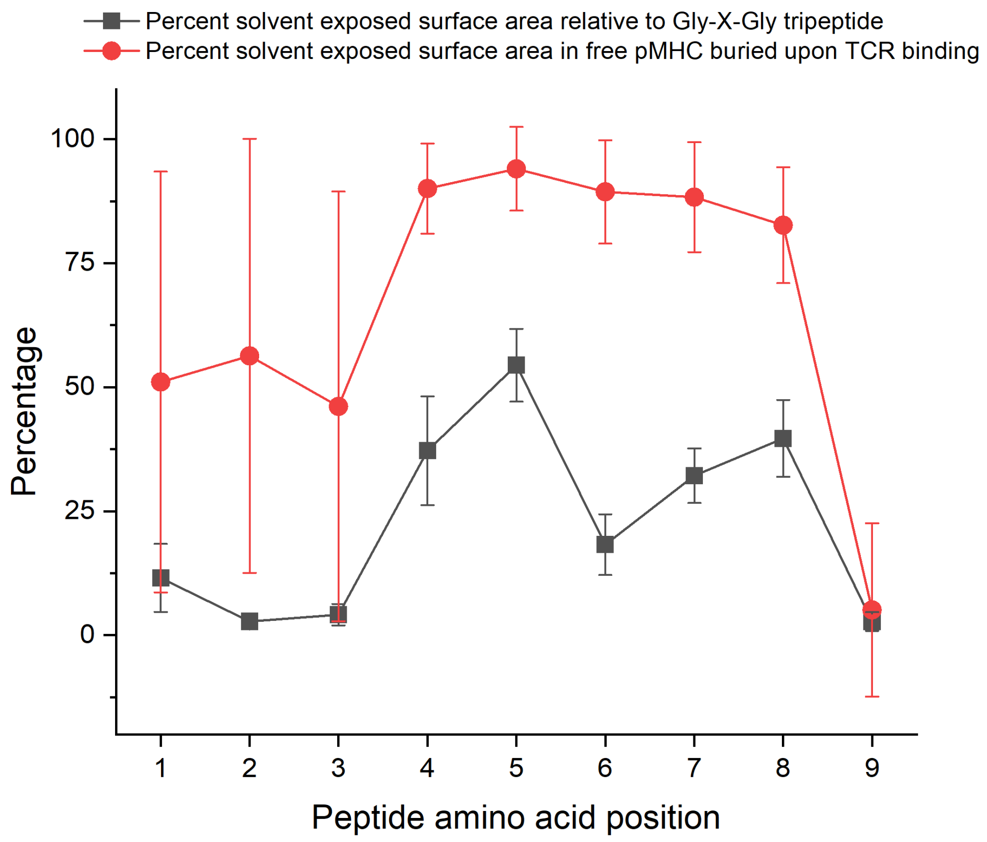

Figure S1. Exposed and buried surface areas for peptides bound to HLA-A2. Black shows the solvent exposed surface area for each peptide side chain for all nonamers in the structures used to benchmark the structural modeling (Table S1) relative to the same amino acid in a Gly-X-Gly extended tripeptide. Red shows the percent of exposed side chain surface area that is buried upon TCR binding for all nonameric TCR-peptide/HLA-A2 structures in the PDB as of October 2018. For both sets of data points indicate averages; error bars indicate standard deviations.

Table S1. Structures utilized in benchmarking structural modeling

		TEMPLATE: 3QFD			TEMPLATE: 1DUZ			TEMPLATE: 5FA3		
**PDB Entry**	**Sequence**	**Energy [a]**	**FA RMSD (Å) [b]**	**Cα RMSD (Å) [c]**	**Energy [a]**	**FA RMSD (Å) [b]**	**Cα RMSD (Å) [c]**	**Energy [a]**	**FA RMSD (Å) [b]**	**Cα RMSD (Å) [c]**
3qfd	AAGIGILTV	-488.09	0.69	0.45	-486.37	2.88	1.71	-525.61	1.79	1.38
1jht	ALGIGILTV	-488.69	0.76	0.35	-489.25	2.77	1.63	-531.82	1.76	1.32
1b0g	ALWGFFPVL	-446.86	3.11	0.96	-504.23	2.10	1.04	-502.78	2.09	0.80
1i7u	ALWGFVPVL	-460.77	3.39	1.86	-505.51	1.87	0.89	-526.15	2.42	0.81
1i7t	ALWGVFPVL	-464.75	2.91	0.98	-504.12	1.65	1.08	-508.03	2.38	1.11
3mrj	CINGMCWTV	-476.58	2.00	0.66	-479.65	1.82	0.83	-527.23	2.07	1.24
3mrg	CINGVCWTV	-471.70	1.68	0.89	-479.91	1.84	1.35	-529.93	1.19	0.72
3mrl	CINGVVWTV	-473.66	1.31	0.83	-483.15	2.16	0.99	-527.94	1.34	1.09
3mrh	CISGVCWTV	-477.16	1.67	0.78	-476.48	2.77	1.13	-524.85	1.56	0.68
2gtw	LAGIGILTV	-484.17	2.59	2.23	-493.51	4.11	3.29	-542.13	3.36	2.95
5hhp	GILEFVFTL	-467.52	1.45	0.31	-492.47	2.16	0.65	-540.14	2.24	0.74
2vll	GILGFVFTL	-467.42	2.35	0.95	-493.32	2.02	0.81	-544.03	2.51	1.32
5hhn	GILGLVFTL	-468.41	1.64	0.72	-490.53	1.61	0.81	-524.80	1.60	0.94
5hhq	GIWGFVFTL	-436.19	1.21	0.52	-512.00	2.81	1.26	-549.38	2.51	0.83
3mrf	GLCPLVAML	-470.61	1.50	0.68	-509.94	1.74	1.18	-546.49	1.30	0.63
3mre	GLCTLVAML	-470.29	1.77	1.05	-505.80	1.47	0.92	-520.30	2.01	1.33
1eez	ILSALVGIL	-472.34	1.85	1.04	-490.17	1.86	1.08	-521.46	1.62	0.95
1eey	ILSALVGIV	-470.09	1.25	0.68	-489.04	1.78	1.00	-533.28	1.51	0.91
1tvh	IMDQVPFSV	-478.66	1.67	0.80	-495.70	3.01	1.78	-529.35	2.48	1.48
1tvb	ITDQVPFSV	-480.64	1.55	0.77	-502.57	3.07	1.82	-526.43	2.39	1.34
3v5h	KVAEIVHFL	-469.46	1.79	0.84	-508.05	1.75	0.86	-528.54	1.66	0.86
3v5d	KVAELVHFL	-434.03	1.59	0.36	-505.42	1.83	0.80	-528.76	2.62	1.23
3v5k	KVAELVWFL	-473.24	2.53	0.88	-485.20	1.45	0.77	-518.27	2.22	0.73
3pwl	LGYGFVNYI	-435.88	3.06	1.29	-490.54	2.30	0.68	-512.14	3.04	1.21
3pwn	LLYGFVNYI	-431.36	2.70	0.91	-498.58	1.03	0.58	-526.20	2.90	1.11
3pwj	LLYGFVNYV	-464.42	2.60	1.43	-501.52	1.03	0.50	-526.29	3.15	1.15
2git	LLFGKPVYV	-433.12	2.34	0.90	-491.56	0.89	0.32	-513.74	2.26	1.35
1im3	LLFGYPVYV	-383.66	2.44	0.69	-494.36	0.80	0.39	-520.81	2.75	1.27
3mrc	NLVPMCATV	-476.86	2.67	1.47	-489.90	2.85	1.67	-540.32	3.00	2.01
3mrd	NLVPMGATV	-472.02	1.20	0.93	-487.46	2.82	1.76	-538.48	2.71	1.91
3gsw	NLVPMVAAV	-469.65	1.52	0.89	-490.86	2.36	1.33	-537.59	1.37	0.51
3gso	NLVPMVATV	-474.97	1.14	0.38	-489.85	2.10	1.17	-539.56	1.07	0.68
3gsx	NLVPMVAVV	-473.99	1.07	0.51	-487.63	2.00	1.15	-538.25	1.13	0.68
3mrb	NLVPMVHTV	-477.22	1.23	0.65	-490.75	1.88	1.24	-535.41	1.16	0.80
3gsv	NLVPQVATV	-475.90	1.39	0.75	-487.62	1.90	1.12	-532.61	1.53	0.73
3gsq	NLVPSVATV	-478.06	1.16	0.65	-489.99	1.75	1.10	-540.76	1.11	0.73
3gsu	NLVPTVATV	-474.65	1.21	0.74	-488.41	1.91	1.24	-538.25	1.31	0.96
3gsr	NLVPVVATV	-465.23	1.07	0.61	-489.64	1.93	1.22	-542.00	1.05	0.70
3mr9	NLVPAVATV	-476.83	1.08	0.70	-490.67	1.83	1.14	-538.78	1.06	0.70
1s8d	SLANTVATL	-475.50	1.33	0.71	-507.98	1.67	0.95	-535.55	1.41	0.85
2v2x	SLFNTVATL	-475.95	2.60	1.49	-509.19	2.40	1.25	-542.41	2.82	1.72
1s9x	SLLMWITQA	-470.59	2.08	1.10	-507.55	2.63	1.48	-541.41	2.04	0.97
1s9w	SLLMWITQC	-470.56	2.34	1.27	-505.68	2.73	1.48	-539.28	2.23	1.12
3kla	SLLMWITQL	-459.05	1.87	0.99	-506.45	2.47	1.42	-544.05	2.32	1.07
1s9y	SLLMWITQS	-469.11	2.07	1.08	-508.22	2.65	1.49	-533.72	1.97	1.01
1t1x	SLYLTVATL	-464.44	2.00	0.60	-504.80	1.76	0.93	-536.73	2.05	0.77
1t20	SLYNTIATL	-469.84	2.11	0.71	-510.30	1.92	0.94	-533.70	1.95	0.81
2v2w	SLYNTVATL	-461.86	2.04	0.63	-508.42	2.01	1.20	-537.16	2.09	0.89
1t1y	SLYNVVATL	-365.42	2.05	0.74	-511.16	1.60	0.87	-535.13	1.99	0.75
3ft3	VLHDDLLEA	-457.06	1.87	0.74	-510.21	2.61	1.21	-534.31	1.56	0.91
3ft4	VLRDDLLEA	-442.43	2.23	1.02	-502.92	2.76	1.20	-538.44	1.38	0.95
3myj	YMFPNAPYL	-394.92	2.23	0.81	-500.13	1.59	1.18	-543.24	2.32	1.16
3hpj	RMFPNAPYL	-463.57	1.85	1.00	-510.67	1.50	1.12	-525.84	1.90	0.91
	Average:	*-462.18*	*1.86*	*0.87*	*-497.08*	*2.08*	*1.15*	*-532.07*	*1.99*	*1.05*
	Stdev	*24.30*	*0.63*	*0.36*	*9.80*	*0.62*	*0.45*	*9.89*	*0.61*	*0.41*

[a] Total energy of modeled peptide/HLA-A2 complex as scored by the Talaris2014 score function in Rosetta energy units

[b] Full atom RMSD of modeled peptide to structure in Ångstroms

[c] α carbon RMSD of modeled peptide to structure in Ångstroms

Table S2. Immunogenic epitopes from the IEDB

Epitope	Source
KVLIRCYLC	Alphapapillomavirus 10
KCIDFYSRI	Alphapapillomavirus 7
NLLIRCLRC	Alphapapillomavirus 7
FAFRDLCIV	Alphapapillomavirus 9
GTLGIVCPI	Alphapapillomavirus 9
KLPQLCTEL	Alphapapillomavirus 9
LLMGTLGIV	Alphapapillomavirus 9
MLDLQPETT	Alphapapillomavirus 9
TLHEYMLDL	Alphapapillomavirus 9
YMLDLQPET	Alphapapillomavirus 9
TLQDVSLEV	Alphapapillomavirus 9
VAANIVLTV	Callithrix jacchus
LPAIVREAI	Dengue virus
AIVREAIKR	Dengue virus
SRNSTHEMY	Dengue virus
TLYAVATTI	Dengue virus
AAAWYLWEV	Dengue virus
GLLTVCYVL	Dengue virus
YLPAIVREA	Dengue virus
KLNDWDFVV	Dengue virus
SMINGVVKL	Dengue virus
CLGGLLTMV	Epstein Barr virus
FLYALALLL	Epstein Barr virus
GLCTLVAML	Epstein Barr virus
LLWTLVVLL	Epstein Barr virus
SLVIVTTFV	Epstein Barr virus
SVRDRLARL	Epstein Barr virus
TLDYKPLSV	Epstein Barr virus
VLQWASLAV	Epstein Barr virus
YLLEMLWRL	Epstein Barr virus
YLQQNWWTL	Epstein Barr virus
YVLDHLIVV	Epstein Barr virus
AFLGERVTL	Epstein Barr virus
FLGERVTLT	Epstein Barr virus
KLGPGEEQV	Epstein Barr virus
RFIAQLLLL	Epstein Barr virus
TLTSYWRRV	Epstein Barr virus
FLFAVGFYL	Epstein Barr virus
FVVPILLKA	Hantaan orthohantavirus
YLSGANLNL	Hepatitis B virus
ALYDVVTKL	Hepatitis C virus
ATDALMTGF	Hepatitis C virus
ATDALMTGY	Hepatitis C virus
CINGVCWTV	Hepatitis C virus

Epitope	Source
FLIAYQPLL	Human herpesvirus 1
RMLGDVMAV	Human herpesvirus 1
TMLEDHEFV	Human herpesvirus 1
ALLGLTLGV	Human herpesvirus 1
FVLATGDFV	Human herpesvirus 1
GIFEDRAPV	Human herpesvirus 1
NLLTTPKFT	Human herpesvirus 1
TMYYKDVTV	Human herpesvirus 1
YLANGGFLI	Human herpesvirus 1
SLYNTIAVL	Human immunodeficiency virus 1
SLYNTVATL	Human immunodeficiency virus 1
SLFNTVATL	Human immunodeficiency virus 1
SLFNAVATL	Human immunodeficiency virus 1
SLFNTIATL	Human immunodeficiency virus 1
SLFNTVAVL	Human immunodeficiency virus 1
SLFNAVAVL	Human immunodeficiency virus 1
SLFNTIAVL	Human immunodeficiency virus 1
SLYNTIAIL	Human immunodeficiency virus 1
SLYNTIATL	Human immunodeficiency virus 1
SLYNTISVL	Human immunodeficiency virus 1
SLYNTVAVL	Human immunodeficiency virus 1
SVYNTVATL	Human immunodeficiency virus 1
SLQQEITLL	Human metapneumovirus
FQANTPPAV	Human metapneumovirus
AIMDKNIIL	Influenza A virus
GILGFVFTL	Influenza A virus
GMLGFVFTL	Influenza A virus
ALPHIIDEV	Lymphocytic choriomeningitis mammarenavirus
SLNQTVHSL	Lymphocytic choriomeningitis mammarenavirus
YLVSIFLHL	Lymphocytic choriomeningitis mammarenavirus
KLWESPQEI	Measles morbillivirus
VLFGLGFAI	Mus musculus
GLPVEYLQV	Mycobacterium kansasii
AVADHVAAV	Mycobacterium tuberculosis
AMASTEGNV	Mycobacterium tuberculosis
KLIANNTRV	Mycobacterium tuberculosis
ALAMEVYQA	Mycobacterium tuberculosis
ALTEMDYFI	Mycobacterium tuberculosis
GINTIPIAL	Mycobacterium tuberculosis
KLEPMASIL	Mycobacterium tuberculosis
AAYPITGKL	Mycobacterium tuberculosis
ATFAAPVAL	Mycobacterium tuberculosis
YLNKIQNSL	Plasmodium falciparum
LLFGYPVYV	Primate T-lymphotropic virus 1

(Table S2 cont)

CVNGVCWTV	Hepatitis C virus
ELIEANLLW	Hepatitis C virus
ETFWAKHMW	Hepatitis C virus
GLQDCTMLV	Hepatitis C virus
ILAGYGAGV	Hepatitis C virus
ILDSFDPLV	Hepatitis C virus
LLCPAGHAV	Hepatitis C virus
SLMAFTAAV	Hepatitis C virus
IMGGELPTA	Hepatitis C virus
KLQVFLIVL	Homo sapiens
LLDVAPLSL	Homo sapiens
LLLLDVAPL	Homo sapiens
SLMSWSAIL	Homo sapiens
FLLDILGAT	Homo sapiens
HIAGSLAVV	Homo sapiens
VVTGVLVYL	Homo sapiens
LLSLFSLWL	Homo sapiens
DLILELLDL	Homo sapiens
FLLFIFKVA	Homo sapiens
KLQQKEEQL	Homo sapiens
LLQEEEEEL	Homo sapiens
NLLEREFGA	Homo sapiens
ILDKKVEKV	Homo sapiens
SLLMWITQC	Homo sapiens
LMLGEFLKL	Homo sapiens
NLVPMVATV	Human cytomegalovirus
VLEETSVML	Human cytomegalovirus
VLGPISGHV	Human cytomegalovirus
LMWYELSKI	Human gammaherpesvirus 8
ALISAFSGS	Human gammaherpesvirus 8
AMLVLLAEI	Human gammaherpesvirus 8
DGGDGNKTL	Human gammaherpesvirus 8
LVLILYLCV	Human gammaherpesvirus 8
PESSQRPPL	Human gammaherpesvirus 8
PVVSTHEQI	Human gammaherpesvirus 8
RLAAGSPSS	Human gammaherpesvirus 8
ILDAHSLYL	Rift Valley fever phlebovirus
VLSEWLPVT	Rift Valley fever phlebovirus
TLLANVTAV	Rotavirus A
LLNYILKSV	Rotavirus A
GLMWLSYFV	Severe acute respiratory syndrome-related coronavirus
RLNEVAKNL	Severe acute respiratory syndrome-related coronavirus
ALLALLIYA	Toxoplasma gondii
FLADLLHSV	Toxoplasma gondii
FLDRALLTL	Toxoplasma gondii
FVGSYLVIV	Toxoplasma gondii
ALDEKLFLI	Vaccinia virus
CLTEYILWV	Vaccinia virus
FLTSVINRV	Vaccinia virus
GLNDYLHSV	Vaccinia virus
ILDDNLYKV	Vaccinia virus
ILSDENYLL	Vaccinia virus
IVIEAIHTV	Vaccinia virus
KIDYYIPYV	Vaccinia virus
KVDDTFYYV	Vaccinia virus
SLSNLDFRL	Vaccinia virus
SMHFYGWSL	Vaccinia virus
YIYGIPLSL	Vaccinia virus
YLIKLIEPV	Vaccinia virus
YVNAILYQI	Vaccinia virus
KLFSDISAI	Vaccinia virus
SLKDVLVSV	Vaccinia virus
TLLDHIRTA	Vaccinia virus
ILIEGIFFV	Varicella-zoster virus
ATWAENIQV	West Nile virus
SLFGQRIEV	West Nile virus
SVGGVFTSV	West Nile virus

Table S3. Structural and structure-derived terms used for training the structure based immunogenicity network. Energetic terms are those that comprise the Talaris2014 energy function and described in detail in ref. 56.

Global energetic terms describing entire peptide/MHC complex		Description
total_score		Total Talaris 2014 total energy
fa_atr		Total Lennard-Jones attractive
fa_rep		Total Lennard-Jones repulsive
fa_sol		Total Lazaridis-Karplus solvation energy
fa_intra_rep		Total Lennard-Jones repulsive between atoms of same residue
fa_elec		Total Coulombic electrostatic potential
pro_close		Total proline ring closure energy
hbond_sr_bb		Total backbone-backbone hydrogen bond energy, close in structure
hbond_lr_bb		Total backbone-backbone hydrogen bond energy, distant in structure
hbond_bb_sc		Total sidechain-backbone hydrogen bond energy
hbond_sc		Total sidechain-sidechain hydrogen bond energy
dslf_fa13		Total disulfide geometry potential
rama		Total Ramachandran preference energy
omega		Total omega dihedral energy in the backbone
fa_dun		Total internal energy of sidechain rotamers as derived from Dunbrack's statistics
p_aa_pp		Total probability of amino acid at phipsi
yhh_planarity		Total torsional potential for Tyrosine
ref		Total of reference energies for each amino acid

Energetic terms at the level of each peptide amino acid		Description
fa_atr		Lennard-Jones attractive (between position atoms and every other atom of pMHC)
fa_rep		Lennard-Jones repulsive (between position atoms and every other atom of pMHC)
fa_sol		Lazaridis-Karplus solvation energy for position
fa_intra_rep	X	Lennard-Jones repulsive between atoms of same residue
fa_elec		Coulombic electrostatic potential (between position and every other atom of pMHC)
rama	X	Ramachandran preferences
fa_dun	X	Internal energy of sidechain rotamers as derived from Dunbrack's statistics
p_aa_pp	X	Probability of amino acid at phi/psi
ref		Amino acid reference energy for position

Additional amino acid level terms (structure dervied, non-energetic)		
sasa		Solvent accessible surface area
hsasa		Hydrophobic solvent accessible surface area

X = excluded after cross-validation

Table S4. Nonameric wild-type and mutant HLA-A2 restricted peptides modeled and examined (see ref. 60)

Wild type peptide	Mutant peptide	Immunogenic (1=yes)	Wild type score	Mutant score
ALALARKGV	ALALAQKGV	0	0.3758	0.2967
ALARKGVQL	ALAQKGVQL	0	0.1808	0.1738
ALAKEFAEI	ALATEFAEI	0	0.3707	0.4443
ALCGQCVRT	ALCGQCVRI	0	0.327	0.5636
ALFLIFSFV	ALFLIFFFV	0	0.9141	0.9489
AVFTWTNLL	ALFTWTNLL	0	0.7611	0.8276
ALIDPSSGL	ALIDLSSGL	0	0.3193	0.4105
TLIGSSSHV	ALIGSSSHV	0	0.4743	0.4609
ALIHHNTHL	ALIHHNTYL	1	0.422	0.5876
ALLMCPLPL	ALLMCLLPL	0	0.5286	0.4552
ALPYFSEMV	ALLYFSEMV	0	0.5598	0.4892
ALMMSLPAL	ALMMSLSAL	0	0.3841	0.3888
ALNLQDFHM	ALNLQDFYM	0	0.2654	0.3445
ALQSRRQAL	ALQSRLQAL	1	0.2829	0.319
ALPAMMQWV	ALSAMMQWV	0	0.7911	0.6016
ALSPVIPLI	ALSPVIPHI	1	0.711	0.5707
ALSACTSHI	ALSTCTSHI	0	0.4661	0.4343
ALGTIWYEL	ALSTIWYEL	0	0.292	0.3105
ALTASPVPL	ALTASTVPL	0	0.3833	0.4188
ALVGAIPSI	ALVGAISSI	0	0.5216	0.5389
ALVVLQELV	ALVPLQWLV	0	0.3923	0.7657
ALYGSVPVL	ALYGFVPVL	0	0.5967	0.8467
AMLKVQEDI	AMFKVQEDI	0	0.1667	0.1918
AMGVAPWGV	AMGVALWGV	0	0.6482	0.6742
AQSKGAWIL	AQFKGAWIL	0	0.2347	0.3939
AGDNFVRFV	AVDNFVRFV	0	0.5081	0.7353
AVGSHVYSV	AVGSYVYSV	1	0.6158	0.672
AVVLALVLL	AVLLALVLL	0	0.6854	0.6906
AVWEYQKPI	AVWEYQKPV	0	0.5809	0.5803
CLSAKLIAI	CLFAKLIAI	0	0.7213	0.8579
CLFLGIYTV	CLFLEIYTV	0	0.5516	0.7148
CVLMLLIPL	CLLMLLIPL	0	0.5496	0.5696
CMQANPHYA	CMQANSHYA	0	0.3538	0.392
IALDRYYSV	FALDRYYSV	0	0.4693	0.4781
SALGNVISA	FALGNVISA	0	0.4696	0.4722
FIDSHTPPL	FIDFHTPPL	0	0.5088	0.4736
IIGPLMDAL	FIGPLMDAL	0	0.42	0.369
FIIPRETLV	FIISRETLV	0	0.5854	0.7263
SILDAVQRV	FILDAVQRV	0	0.299	0.3214
FILEIINAV	FILEIINAL	0	0.5896	0.5092
SIVEALTLL	FIVEALTLL	0	0.5474	0.4741
FLATSGVPV	FLATLGVPV	0	0.194	0.4737
FPEEMATYL	FLEEMATYL	0	0.3468	0.5219
FLGIYTVTV	FLEIYTVTV	0	0.6727	0.7089

(Table S4 cont)

FSFAISVTT	FLFAISVTT	0	0.5648	0.6064
FLFAAVVAW	FLFDAVVAW	0	0.8013	0.6033
FLFLFFVLF	FLFFCFVLF	0	0.7498	0.6942
FLFHTSLPI	FLFHTFLPI	0	0.6234	0.7718
FLFILIQLV	FLFIVIQLV	0	0.6897	0.6427
FLGLGLSGI	FLFLGLSGI	0	0.3505	0.3986
FSFSGVNRL	FLFSGVNRL	0	0.226	0.2538
FLGLTPVGV	FLGLTLVGV	0	0.4535	0.4787
LLIAFGHRV	FLIAFGHRV	0	0.7858	0.8894
FLIPIVNIS	FLIPIVFIS	0	0.4065	0.71
FLTYLDVSV	FLIYLDVSV	1	0.5495	0.5194
FLLALHDSV	FLLALYDSV	0	0.3862	0.3703
FLQGKTSSV	FLLGKTSSV	0	0.5321	0.5302
SLLILFAEI	FLLILFAEI	0	0.6215	0.6876
FLLLVVAMI	FLLLVAAMI	0	0.8906	0.898
FLLQYPEVA	FLLQYSEVA	0	0.2891	0.3384
FPLTSIIAI	FLLTSIIAI	0	0.4956	0.6689
FLLYNLGSV	FLLYNLGLV	0	0.3326	0.4448
FLMASISSS	FLMASISSF	0	0.2989	0.4127
FVNDIFERI	FLNDIFERI	0	0.3934	0.2803
FLNEIPPFV	FLNEIPLFV	0	0.6101	0.6318
FLNMELLVE	FLNMELLVV	0	0.1104	0.5908
FLNSCFSCI	FLNSCFFCI	0	0.6123	0.812
SLPAYLNSL	FLPAYLNSL	0	0.4679	0.4521
FLQSRGNEV	FLQFRGNEV	0	0.1821	0.1752
FLQEYTVKL	FLQKYTVKL	0	0.4616	0.5449
SLQPHMIGA	FLQPHMIGA	0	0.4679	0.4951
FLRQRVAFL	FLSQRVAFL	0	0.6543	0.4684
SLVCVVPDV	FLVCVVPDV	0	0.3388	0.3395
SMPDFDLHL	FMPDFDLHL	1	0.5084	0.5321
FMSFQSYFL	FMSFQSYLL	0	0.3238	0.4998
FMSSHIKSV	FMSSHMKSV	0	0.3429	0.2844
FMSTWFLLV	FMSTRFLLV	0	0.7952	0.5404
SQLNQSFEI	FQLNQSFEI	0	0.2137	0.162
FRNYKQVPV	FQNYKQVPV	0	0.2078	0.1113
FQPSFPHLV	FQPSFSHLV	0	0.6626	0.6989
FSFPLDFLV	FSFSLDFLV	0	0.8369	0.7487
FPGEYIPTV	FSGEYIPTV	0	0.2721	0.268
FTLVGVWLV	FTLVEVWLV	0	0.853	0.9124
GNILDVPEI	GIILDVPEI	0	0.1748	0.3885
GLDLSTCPI	GLDLSTFPI	0	0.3343	0.5155
GLEFQISNV	GLEFQIFNV	0	0.2496	0.3714
GSFGDIYLA	GLFGDIYLA	0	0.532	0.5876
GLLHGCIHA	GLLHGCIYA	0	0.3215	0.3098
GLMSMMIPL	GLMFMMIPL	0	0.5763	0.4947

(Table S4 cont)

GLMKDIVGA	GLMKDIVEA	0	0.3836	0.2369
GLDETIAKL	GLNETIAKL	0	0.4291	0.3713
GPPHAFFLV	GLPHAFFLV	0	0.562	0.8262
GLSWLCSPL	GLSWLPPPL	0	0.2711	0.3989
GLVDEQQKV	GLVDEQQEV	0	0.2453	0.2155
GSVEALHEV	GLVEALHEV	0	0.2263	0.2418
GMTPCLLAL	GMPPCLLAL	0	0.4573	0.5823
GSLDVPMAV	GSLDVLMAV	0	0.5865	0.5564
GVINAAFML	GVINAEFML	0	0.733	0.427
GVINSFVHV	GVISSFVHV	0	0.4761	0.5467
GVWALPGPI	GVWALPDPI	0	0.8922	0.7799
RLFCVFYGL	HLFCVFYGL	0	0.6868	0.6907
NLMDGDLGL	HLMDGDLGL	0	0.2813	0.2949
HLTCLLLWV	HLTCLLLWV	0	0.8988	0.8698
NILDVPEIV	IILDVPEIV	0	0.3738	0.5117
IINAVPFII	IINALPFII	0	0.9607	0.9113
IINRTPGSL	IINRTPWSL	0	0.3241	0.7824
IWAALFLGL	ILAALFLGL	1	0.8045	0.742
ILAFVGAAV	ILAFVAAAV	0	0.6994	0.7996
ILGVCSSGL	ILDVCSSGL	0	0.2633	0.3192
HLFLFFLFV	ILFLFFCFV	0	0.9421	0.9313
ILFLIISSV	ILFLIISYV	0	0.8074	0.9176
IPGNALPLV	ILGNALPLV	0	0.3838	0.4848
ILILYLLTI	ILILYLLSI	0	0.7823	0.8146
IPIPSTGSV	ILIPSTGSV	0	0.3149	0.4138
ILSDELKPA	ILLDELKPA	0	0.2644	0.2943
TLLDSTWNL	ILLDSTWNL	0	0.3389	0.3303
ILLSFENHV	ILLLFENHV	0	0.3937	0.406
ILLMTVISI	ILLMTVTSI	0	0.8728	0.728
ILPTCEPTV	ILLTCEPTV	0	0.4082	0.3831
ILLTLVAIL	ILLTLVANL	0	0.8558	0.5427
ILMEHIHKL	ILMEHSHKL	0	0.4951	0.4493
ILNAMITKI	ILNAMIAKI	1	0.7058	0.8008
ILSTLPYTM	ILSTLPYTI	0	0.3923	0.6792
ILTGLNYEA	ILTGLNYEV	1	0.3283	0.4019
ILDEAYVMA	ILYEAYVMA	0	0.3233	0.4323
IMAPANSFV	IMAPANFFV	0	0.2752	0.4686
KAFESGIPV	KAFELGIPV	0	0.2868	0.5189
KINLSLSAL	KINLSLFAL	0	0.3831	0.628
KSFEQFSKL	KLFEQFSKL	0	0.2269	0.288
KSFSQIYSL	KLFSQIYSL	0	0.3882	0.4635
KLIFHRHPV	KLIFHRHSV	0	0.442	0.4667
KLVMSQANV	KLLMSQANV	0	0.1876	0.1756
KLLPTNTDI	KLLPTNTNI	0	0.2456	0.2568
KLMDGTSQA	KLMDGTSQV	0	0.16	0.1882

(Table S4 cont)

KSMNQAICI	KLMNQAICI	0	0.3231	0.6106
KLNKYHDWL	KLNKYYDWL	0	0.3887	0.363
KLQPLSPVP	KLQPLSPVV	0	0.4297	0.5229
ELQQLLFSV	KLQQLLFSV	0	0.4662	0.5825
KLSHQPVLL	KLSHQLVLL	1	0.326	0.4243
ELYALNENI	KLYALNENI	0	0.2518	0.2515
EMAESLLQV	KMAESLLQV	0	0.1446	0.1489
KMTFLFANL	KMTFLFPNL	0	0.1623	0.1546
KQHFINQAV	KQQFINQAV	0	0.175	0.2076
KVWCCQILL	KVCCCQILL	1	0.7112	0.4765
KIDFTEFLL	KVDFTEFLL	0	0.6266	0.44
KVDIFSLGL	KVDIFFLGL	0	0.651	0.845
KVFSWSSYL	KVFSCSSYL	0	0.8069	0.4386
LIIPCIHLI	LIIPFIHLI	1	0.8408	0.9714
LIISSVASL	LIISYVASL	0	0.6066	0.8382
LLAKRQVAL	LLAKREVAL	0	0.3502	0.3356
VLALVLLLL	LLALVLLLL	0	0.7384	0.6102
LLAWMIPDV	LLAWMIPNV	0	0.2443	0.3214
LLCTDPGFV	LLCADPGFV	0	0.4042	0.5345
LLDSLPMDA	LLDSLPMDV	0	0.2896	0.3945
LLFGMTPCL	LLFGMPPCL	1	0.6892	0.6925
LLFSITGFL	LLFSETGFL	0	0.7842	0.5672
LLGVKLSGV	LLGVKLFGV	0	0.3722	0.6976
LLHMGQPDL	LLHMCQPDL	0	0.1607	0.1557
LLIHCQAGV	LLIYCQAGV	0	0.5139	0.4411
PLKDCIFDL	LLKDCIFDL	0	0.4668	0.2282
LLLAGAPAA	LLLAGVPAA	0	0.4431	0.4312
LLLAVTPWV	LLLAVTSWV	0	0.8864	0.8218
LLLSFSVWI	LLLFFSVWI	0	0.8896	0.8888
LLLQVLFRL	LLLQVLFWL	0	0.5053	0.7825
LLLSARVYA	LLLSARFYA	0	0.386	0.467
LPLSEHWAL	LLLSEHWAL	0	0.6164	0.7899
LLLPISLLI	LLLSISLLI	0	0.84	0.7886
SLMPSLENL	LLMPSLENL	0	0.1968	0.1726
LLRVHTEQV	LLRVHTEHV	0	0.2977	0.3663
LLSIDLFHV	LLSIDLFYV	0	0.4528	0.5088
LLSPLGAEV	LLSPLGAEI	0	0.271	0.3955
LLTTIYIFL	LLTTIYFFL	0	0.5459	0.5996
LPVAAEPYL	LLVAAEPYL	0	0.3337	0.3542
LLVLGQQPV	LLVLCQQPV	0	0.1897	0.2319
LLVLWALCV	LLVLWAFCV	0	0.9402	0.9442
PLVVKFCNV	LLVVKFCNV	0	0.6398	0.4052
LMASISSSL	LMASISSFL	0	0.2946	0.4939
LMFAISFLT	LMFAIFFLT	0	0.8186	0.7299
LPALLLQHV	LQALLLQHV	0	0.2865	0.1817

(Table S4 cont)

LFFGCVFWL	LVFGCVFWL	0	0.8843	0.9371
LVIGRIFHI	LVIGRIFYI	0	0.8456	0.8632
MLCAGNPEV	MLCAENPEV	0	0.1817	0.2646
MLFLRFRYI	MLFLRFCYI	0	0.5035	0.7513
MLWGSSTQL	MLWGSLTQL	0	0.4621	0.4508
MLYVVPRPG	MLYVVPWPG	0	0.3136	0.817
MLNPLIYSL	MMNPLIYSL	0	0.5063	0.4267
NLFFVFYML	NLIFVFYML	0	0.6316	0.6844
NLLRILNKL	NLLRILYKL	0	0.3219	0.4372
NLNRCSVPV	NLNCCSVPV	1	0.3039	0.4383
NLQDFHMHL	NLQDFYMHL	0	0.4529	0.4625
NLYRVHFPV	NLYRVYFPV	0	0.6012	0.5676
HLFEDAYLL	QLFEDAYLL	0	0.5759	0.4606
QLLARLTGV	QLLAKLTGV	0	0.603	0.619
QLMQLIGPA	QLMQLIEPA	0	0.3692	0.2124
QLSLGIIPV	QLSLGIILV	0	0.2749	0.4702
QQFAVGSHV	QQFAVGSYV	1	0.2171	0.2569
QQQEATPPA	QQQEATPPV	0	0.1282	0.132
RIFLLIQSV	RIFLLIQFV	0	0.5903	0.7998
RLFFENHPA	RLFFENPPA	0	0.3148	0.3155
RRFHELEAI	RLFHELEAI	0	0.2041	0.3334
RLGEHNIEV	RLGEHNIEV	0	0.1151	0.1299
RLRWWQPFV	RLGTWMPFV	0	0.7684	0.7526
RLLEEVEEL	RLLEEVEEL	0	0.1703	0.1506
RLMFAISFL	RLMFAIFFL	0	0.3621	0.6103
RRQALGPEA	RLQALGPEA	0	0.1048	0.2392
RLQASPFRL	RLQASLFRL	0	0.3171	0.4185
RMFPTPPSL	RMFSTPPSL	0	0.3014	0.2391
RMLDKNPES	RMLDKNPEV	0	0.1076	0.1612
RMTRYLESW	RMTRYLESL	0	0.2276	0.1849
SIPAFLYFL	SISAFLYFL	0	0.9508	0.8992
SIWGFSGNA	SIWGFSGNV	0	0.7292	0.8002
SLAAGVWGL	SLAACVWGL	0	0.6852	0.86
SLAPLSPRA	SLAPLSPRV	0	0.3759	0.4416
SLDPTTSPV	SLDLTTSPV	0	0.3583	0.4383
SLEEKDQYI	SLEEKDHYI	0	0.2459	0.5079
SLFGFSFNA	SLFRFSFNA	0	0.6622	0.5666
SPFRLVPNV	SLFRLVPNV	0	0.2853	0.4642
SLFTANSHL	SLFTANFHL	0	0.3249	0.45
SLGTFVSYL	SLGTFVFYL	0	0.6939	0.852
SLIHSTAQI	SLIHYTAQI	0	0.4269	0.4976
SLILVENPV	SLILLENPV	0	0.3323	0.3508
SLISEQWVV	SLISEQWVV	0	0.7352	0.7525
SLLALASLA	SLLALASLV	0	0.8077	0.8392
SLLALVHVV	SLLALVHVV	0	0.9046	0.9036

(Table S4 cont)

SPLMNIEVV	SLLMNIEVV	0	0.225	0.2937
SSLSLLYAL	SLLSLLYAL	0	0.6533	0.7525
SLMEPWALG	SLMEHWALG	0	0.4658	0.4693
SLSELRRLV	SLSELQRLV	0	0.4683	0.3391
SLPIGSAEV	SLSIGSAEV	0	0.2631	0.1878
SSSSVTLLL	SLSSVTLLL	0	0.592	0.6955
SLSTSSSSV	SLSTSLSSV	0	0.282	0.2808
SVSINTFPV	SMSINTFPV	0	0.4974	0.4131
SQVYLIVQV	SQVYLILQV	0	0.1552	0.1799
STASDFLAV	STAFDFLAV	0	0.4348	0.4282
ATMSFGILL	STMSFGILL	0	0.8575	0.8305
SVVDVFSQL	SVVDVFFQL	0	0.2817	0.3294
TLAYPFQSL	TLAYSFQSL	0	0.2106	0.2077
TLFPLPFLI	TLFPLPFFI	0	0.9448	0.9408
TLFTPSYGV	TLFTLSYGV	0	0.6731	0.801
TLLGLSLQV	TLLSLSLQV	0	0.6191	0.5768
TLSDVLSRL	TLSDVLFRL	0	0.2833	0.3862
TPWCSPIKV	TLWCSPIKV	1	0.6439	0.7514
TTDDDNLEL	TMDDDNLEL	0	0.1665	0.1782
TQFRCAVAL	TQFWCAVAL	0	0.2199	0.3241
TVQDLQGEV	TVQDLQVEV	0	0.3024	0.319
VILSYTYII	VIFSYTYII	0	0.9453	0.9538
VILELLTPL	VILELLTPL	0	0.5414	0.585
VLAFLVHEL	VLAFFVHEL	0	0.3792	0.5397
VLGSGAFGT	VLASGAFGT	0	0.2995	0.3037
VSDEDFSAL	VLDEDFSAL	0	0.2954	0.317
VLDRESPFV	VLDREFPFV	0	0.3878	0.4496
VLSQTHTNV	VLFQTHTNV	0	0.2346	0.3575
VLFRLITFV	VLFWLITFV	0	0.8605	0.9538
VVLALVLLL	VLLALVLLL	0	0.93	0.8995
VLLLLLLGV	VLLLLLAGV	0	0.8212	0.803
VLLWEIFSL	VLLWEIFFL	0	0.7764	0.8811
VLMGHVAAV	VLMGYVAAV	0	0.7986	0.8569
VPSDNVVMV	VLSDNVVMV	0	0.4443	0.5088
VLWALCVGL	VLWAFCVGL	0	0.9018	0.9278
VLYGVTPSL	VLYGVTLSL	0	0.551	0.6486
VMDPIDVKI	VMDSIDVKI	0	0.3902	0.4982
VMKFKNPPV	VMKFKNPLV	0	0.4579	0.585
VMLKYLFPL	VMLKYLFSL	0	0.6563	0.6701
VTFELTGET	VTFELTWET	0	0.164	0.3149
VTMSWKVRV	VTMSWKERV	0	0.8737	0.4645
VTNSGKFLI	VTYSGKFLI	0	0.6287	0.7194
VVLSWAPPV	VVMSWAPPV	1	0.8745	0.8226
WLIDMESLV	WLIDMKSLV	0	0.5532	0.6106
RLITFVLNA	WLITFVLNA	0	0.5955	0.7112

(Table S4 cont)

WLMPPTIPL	WLMPLTIPL	0	0.3247	0.4767
WMAGFLIAV	WMAEFLIAV	0	0.8197	0.5771
WMSDSGTGL	WMSDSGTRL	0	0.2031	0.1584
HIVENDLYI	YIVENDLYI	0	0.401	0.4455
YIVSNRMVV	YIVSNCMVV	0	0.7089	0.7947
YLDELIKNT	YLDELIRNT	0	0.1556	0.1656
YLDNRFFTL	YLDNRFFTL	0	0.6506	0.5967
YLDHIKHPM	YLDYIKHPM	0	0.3352	0.3749
YPLDFTFVC	YLLDFTFVC	0	0.6749	0.8338
YLLHENCML	YLLHKNCML	0	0.4553	0.603
HLLLWLSPI	YLLLWLSPI	0	0.8123	0.7621
YFLQKTMPV	YLLQKTMPV	0	0.5735	0.5359
YLLTILGNT	YLLSILGNT	0	0.2091	0.2383
FLMKGPNKI	YLMKGPNKI	0	0.2943	0.2468
YLQEKSCMA	YLQEKTCMA	0	0.516	0.431
YLRSVPAVV	YLRSVPAGV	0	0.8157	0.8155
YLSPSMIVI	YLSPFMIVI	0	0.3994	0.7314
YLVDSVAKT	YLVDSVAKM	0	0.2525	0.3152
YLVLFFYPL	YLVLFFYLL	0	0.6334	0.7952
YLVSFFEGL	YLVSFFEGI	0	0.3672	0.4126
YTAPHPHPA	YTAPYPHPA	0	0.2557	0.3183
YTPEHAASV	YTSEHAASV	0	0.5825	0.3155

Supplemental File 1. PyRosetta modeling script used for modeling nonameric peptides bound to HLA-A2.

```
'''
peptide_MHC-modeling.py
PyRosetta4 script for predictive structural modeling of
nonameric peptide-MHC structures.
Easily adaptable to other peptide lengths.

Usage: python peptide_MHC-modeling.py template peptide n

where template is the starting structure in PDB format
peptide is the nonameric peptide to be modeled
and n is the number of decoys to generate.

It is assumed that the peptide is present in the template model as
a separate chain, designated chain C.
-- GLJ Keller and TP Riley
'''
# stdlib imports
from __future__ import print_function
import os
from sys import argv
from random import randint
# PyRosetta4 initialization, enabling use of talaris scorefunctions
from pyrosetta import * ; from pyrosetta.rosetta import *
init(extra_options = "-extrachi_cutoff 12 -ex1 -ex2 -ex3 -corrections::restore_talaris_behavior")
# PyRosetta utility imports
from pyrosetta.toolbox import mutate_residue

_, template, peptide, n = argv

template_model = pose_from_pdb(template)
scorefxn = create_score_function('talaris2014')
positionlist = range(template_model.pdb_info().pdb2pose('C',1),template_model.pdb_info().pdb2pose('C',9)+1)

# create separate output directory for each peptide modeled
output_dir = '_models_{0}'.format(peptide)
if not os.path.exists(output_dir): os.mkdir(output_dir)
os.chdir(output_dir)

# PyRosetta job distributor to parallelize modeling
jd = PyJobDistributor(peptide, int(n), scorefxn)
jd.native_pose = template_model

while not jd.job_complete:
	# assign starting model to new pose for later comparison
	mutant = Pose() ; mutant.assign(template_model)
	# mutate peptide residue in template structure at position i to res for each position in peptide
	#	(assumed chain C)
	for i, res in enumerate(peptide):
		mutate_residue(mutant, positionlist[i], res, 0.0, scorefxn)
	# assign mutant model (with starting coordinates) to new pose for later comparison
	remodel_target = Pose() ; remodel_target.assign(mutant)
	# define loop peptide_ft to peptide residues p2 through p9, with the cutsite set randomly per decoy
	peptide_ft = rosetta.protocols.loops.Loop(positionlist[1], positionlist[7], positionlist[randint(2, 6)])
	peptide_loops = rosetta.protocols.loops.Loops() ; peptide_loops.add_loop(peptide_ft)
	# define fold tree of model as peptide_ft object
	rosetta.protocols.loops.set_single_loop_fold_tree(remodel_target, peptide_ft)
	# create repacking task; disallow changing residue identity; allow repacking to current rotamer
	task_pack = rosetta.core.pack.task.TaskFactory.create_packer_task(remodel_target)
	task_pack.restrict_to_repacking() ;   task_pack.or_include_current(True)
	pack = rosetta.protocols.minimization_packing.PackRotamersMover(scorefxn, task_pack)
	# repack model after mutation
	pack.apply(remodel_target)
	# use Cyclic Coordinate Descent algorithm to remodel peptide coordinates with flexible backbone
	loops_refine_CCD = rosetta.protocols.loops.loop_mover.refine.LoopMover_Refine_CCD(peptide_loops, scorefxn)
	loops_refine_CCD.max_inner_cycles(10)
	loops_refine_CCD.apply(remodel_target)
	# finally, repack and output final model
	pack.apply(remodel_target)
	jd.output_decoy(remodel_target)
```
